# CARING FOR PERSONS LIVING WITH PRIMARY PROGRESSIVE APHASIA: AN INVESTIGATION OF THE MALE EXPERIENCE

**DOI:** 10.1093/geroni/igae098.0124

**Published:** 2024-12-31

**Authors:** Lauren Dowden, Darby Morhardt, Kaitlyn Lucca

**Affiliations:** Northwestern University Feinberg School of Medicine, Chicago, Illinois, United States; Northwestern University, Evanston, Illinois, United States; Northwestern University, Evanston, Illinois, United States

## Abstract

Primary progressive aphasia (PPA) is a clinical syndrome that begins with language difficulties at symptom onset and for the initial phases of the disease. Multiple domains are affected over time, including behavior and personality changes. There are heterogeneous neuropathologic causes; namely, frontotemporal lobar degeneration or Alzheimer’s disease. While there is evidence that women comprise the majority of caregivers for persons living with dementia, little is known regarding sex and gender differences in the experience of caregiving. Studies on caregivers in persons with PPA are rare and none have explored gender differences in the PPA caregiving role. This study explores the male experience of caring for persons living with PPA analyzing in depth interviews from a sample of 17 male caregiving spouses who attend a monthly PPA care partner support group. Open-ended questions explore coping strategies, approaches to the caregiving role, help-seeking behaviors, use of services, and overall caregiver experience. This analysis describes the male experience of caregiving for persons with PPA, highlights their unique needs over the illness course and provides a nuanced understanding of the gendered caregiver role within the context of a specific clinical profile versus broader dementia categories.

